# Unraveling the roles of CD44/CD24 and ALDH1 as cancer stem cell markers in tumorigenesis and metastasis

**DOI:** 10.1038/s41598-017-14364-2

**Published:** 2017-10-23

**Authors:** Wenzhe Li, Huailei Ma, Jin Zhang, Ling Zhu, Chen Wang, Yanlian Yang

**Affiliations:** 1 CAS Key Laboratory of Standardization and Measurement for Nanotechnology, CAS Key Laboratory of Biological Effects of Nanomaterials and Nanosafety, CAS Center for Excellence in Nanoscience, National Center for Nanoscience and Technology, Beijing, 100190 P.R. China; 2 Academy for Advanced Interdisciplinary Studies, Peking University, Beijing, 100871 China; 3 University of Chinese Academy of Sciences, 19 A Yuquan Rd, Shijingshan District, Beijing, P.R. China 100049

## Abstract

CD44/CD24 and ALDH1 are widely used cancer stem cell (CSC) markers in breast cancer. However, their expression is not always consistent even in the same subtype of breast cancer. Systematic comparison of their functions is still lacking. We investigated the expression of CD44, CD24 and ALDH1 in different subtypes of breast cancer cells, and explored their relationship with cancer progression. We defined a parameter CD44/CD24 ratio to present the expression level of CD44 and CD24 and found that high CD44/CD24 ratio and ALDH1^+^ are both indicators for cancer malignancy, but play different functions during tumor progression. High CD44/CD24 ratio is more related to cell proliferation and tumorigenesis, which is confirmed by mammosphere formation and tumorigenesis in xenotransplanted mice. ALDH1^+^ is a stronger indicator for cell migration and tumor metastasis. Suppression of CD44 and ALDH1 by siRNA led to decreased tumorigenicity and cell migration capacity. The combination of high CD44/CD24 ratio and ALDH1^+^ would be a more reliable way to characterize CSCs. Moreover, both high CD44/CD24 ratio and ALDH1^+^ were conserved during metastasis, from the primary tumors to the circulating tumor cells (CTCs) and the distant metastases, suggesting the significant value of these CSC markers in assisting cancer detection, prognostic evaluation, and even cancer therapeutics.

## Introduction

Tumors are heterogeneous due to the contribution of clonal evolution, microenvironmental differences, and the hierarchical organization as a result of differentiation from tumorigenic cells into non-tumorigenic cells^1,2^. The tumor-initiated cancer cells are termed cancer stem cells (CSCs) which are defined as a subpopulation of tumor cells with the capacity for self-renewal and differentiation to drive the initiation, progression, metastasis and recurrence of tumor^3–5^. Since the proposal of CSC hypothesis, a growing body of evidence has demonstrated the existence of these stem-like/progenitor cells in leukemia^6^ and various solid tumors such as breast^7^, brain^8^, colon^9^, liver cancers^10^ and melanoma^11^, and has proved their association with poor prognosis^12^. CSCs exhibit anti-cancer treatment resistance that can avoid being killed by conventional chemo- and radio-therapies, as well as the properties to remain viable and to enable the re-establishment of tumors^13^. Therapeutic strategies targeting CSCs hold great potential in inaugurating a new era in cancer treatment^14^. Therefore, to identify and characterize cancer cells with stemness is essential for the prognostic evaluation and cancer therapy.

The most common way to identify CSCs is through investigating the expression of characteristic cell surface markers. High expression of CD44 and low expression of CD24 (CD44^+^/ CD24^−/low^) is one of such markers. In breast cancer, the CD44^+^/CD24^−/low^ cells from patients were found to be more tumorigenic than the CD44^+^/CD24^+^ cells when implanted into the mammary fat pads of the immunodeficient nonobese diabetic (NOD)/severe combined immunodeficient (SCID) mice^7^. Although the relationship between CD44^+^/CD24^−/low^ and the clinical outcome is not certain, breast tumors with expression of CD44^+^/CD24^−/low^ have been shown to exhibit enhanced invasion and metastasis^15,16^. As stem-like/progenitorial functions have been conserved in CSCs, other functional markers such as aldehyde dehydrogenase 1 (ALDH1) are also widely used to characterize stemness. ALDH1 is a detoxifying enzyme responsible for the oxidation of retinol to retinoic acid which is essential for the early differentiation of stem cells^17^. Increased ALDH1 activity has been found in normal and malignant stem/progenitor breast cells, and can serve as an indicator for poor prognosis^18^. However, the expression of these well-established stem markers does not always correlate with each other. Studies have shown that CD44/CD24 and ALDH1 expressed differently in different subtypes of breast cancers. The CD44^+^/CD24^−/low^ phenotype is more associated with basal-like breast cancers, while the ALDH1^+^ cells are more common in HER2-overexpression (HER2-OE) and basal/epithelial breast cancers^19,20^. Moreover, it has been found that only a fraction of CD44^+^/CD24^−/low^ breast cancer cells were ALDH1 positive, and these cells were more tumorigenic compared to the ALDH1 negative population^18,21^. The mechanism underlying the different expression of CD44/CD24 and ALDH1 in breast cancer has yet to be found. Systematic study on the biological functions of these CSC markers is still lacking.

On the other hand, the correlation between the expression of stem markers and the invasive properties and metastatic potential of tumors has been generally accepted^16,22^. The expression of CD44^+^/CD24^−/low^ and ALDH1^+^ has been revealed in the axillary lymph node metastases of breast cancer^23–26^. As disseminated tumor cells (DTCs) or circulating tumor cells (CTCs) are considered as a subset of cancer cells that transit through the bloodstream from the primary tumor to the metastases, one would expect that the stem markers might be also conserved in these cells. This hypothesis has been confirmed in several recent studies showing the expression of stem markers in the bone marrow^27,28^ and peripheral blood^29^ of breast cancer patients. Nevertheless, whether the stem markers are stable and how their expression changes during the whole process of metastasis are still unknown. Systematic investigations on the expression of stem markers in the primary tumor, CTCs and the distant metastases are scarce.

In the present study, we systematically investigated the expression of CD44, CD24 and ALDH1 in different subtypes of breast cancer cell lines, and explored their possible roles during cancer progression both at the cellular level and in the xenotransplanted mice model. We found that both high CD44/CD24 ratio and ALDH1^+^ correlated with tumor malignancy. However, these two stem markers expressed differently in different subtypes of breast cancer, and had different functions in tumor progression. High CD44/CD24 ratio was more related to cell proliferation and tumorigenesis, while ALDH1^+^ was a stronger indicator for metastasis. Single CSC marker alone can not characterize stem properties. The combination of high CD44/CD24 ratio and ALDH1^+^ may be a more accurate and reliable way to refine the definition of CSCs in breast cancer. Furthermore, both markers showed conserved expression in the primary tumor, CTCs, and the distant metastases, suggesting that they were stable during the development and metastasis of breast cancer. Considering the commonness of these stem markers in various cancers, this combination of markers could therefore serve as valuable biomarkers to monitor tumor progression and to predict prognosis.

## Results

### High CD44/CD24 ratio and ALDH1^+^ correlate with breast cancer malignancy

**Figure 1:**
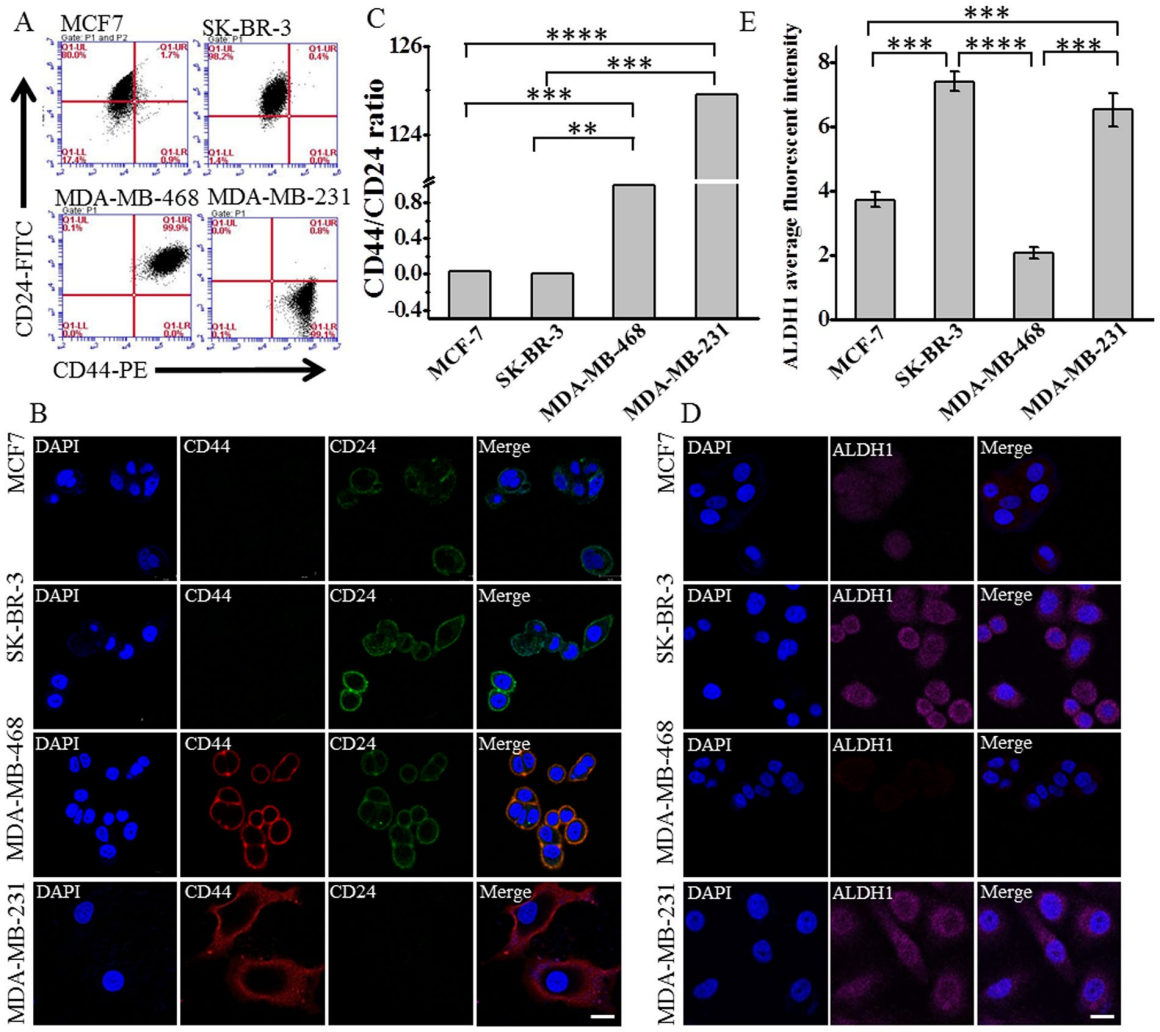
The expression of CD44, CD24 and ALDH1 in different molecular subtypes of breast cancer. Four breast cancer cell lines were compared: MCF-7 (Luminal A subtype), SK-BR-3 (HER2 over expression subtype), MDA-MB-468 (basal-like epithelial subtype), and MDA-MB-231 (basal-like mesenchymal subtype). (**A**) Flow cytometry analysis of the expression of CD44 and CD24 in different breast cancer cell lines. Cells were double stained with anti-CD44-PE and anti-CD24-FITC. The threshold lines were set according to the isotype control. The accuracy of the double immunostaining was confirmed by comparison with single immunostaining for CD44 and CD24 respectively. (**B**) Immunofluorescence images showing the expression of CD44 and CD24 in different subtypes of breast cancer cell lines. Cells were labeled with anti-CD44-PE, anti-CD24–FITC and the nuclei stain DAPI. Scale bar: 20 μm. (**C**) The ratio of CD44/CD24 in different subtypes of breast cancer cell lines. Ratios were calculated from the percentage of CD44 and CD24 positive subpopulations in the flow cytometry analysis. Data represent means ± SD (n = 3), **P < 0.01, ***P < 0.001, ****P < 0.0001. (**D**) Immunofluorescence images showing the expression of ALDH1 in different breast cancer cell lines. Cells were labeled with anti-ALDH1 (magenta) and DAPI (blue), scale bar: 20 μm. (**E**) The average fluorescent intensities of ALDH1 in different breast cancer cell lines, data were calculated from three parallel immunofluorescence images. Data represent means ± SD (n = 3), ***P < 0.001, ****P < 0.0001.

It has been widely accepted that breast cancer is heterogeneous at both morphological and genetic level^30,31^. Normally, breast cancer can be classified into four major molecular subtypes: luminal A and B, HER2-OE and basal-like^32,33^. Different subtype exhibits different malignancy, metastatic potential and treatment resistance^32,34,35^. Generally, patients with basal-like tumors have poorer prognosis whereas those with luminal A tumors have more favorable outcome^32,36,37^. The molecular mechanism underlying their different aggressive behavior is still elusive. To investigate the stem properties of different subtype, we compared the expression of CD44, CD24 and ALDH1 in four human breast cancer cell lines using flow cytometry analysis and immunostaining: MCF-7 (luminal A), SK-BR-3 (HER2-OE), MDA-MB-468 (basal epithelial), and MDA-MB-231 (basal mesenchymal, triple-negative)^32,33^. As expected, the most malignant basal mesenchymal cell line MDA-MB-231 mainly showed CD44^+^/CD24^−^ feature (Fig. 1A,B), while the other three cell lines did not, in accordance with the previous findings showing that CD44^+^/CD24^−/low^ is a stem-like marker highly related to the malignance of breast cancer^19,38,39^. We also found that the luminal A cell line MCF-7 and the HER2-OE cell line SK-BR-3 were mainly composed of cells bearing the CD44^-^/CD24^+^ phenotype, while the basal epithelial cell line MDA-MB-468 mainly showed CD44^+^/CD24^+^ (Fig. 1A,B). However, it is difficult to evaluate the malignancy of these cell lines only based on the traditional stem marker CD44^+^/CD24^−^. Therefore, we further calculated the ratio of the expression level of CD44 and CD24 (CD44/CD24) from the percentage of CD44 and CD24 subpopulations in the flow cytometry analysis (Supplementary Table S1), and found that the CD44/CD24 ratio is the highest in the basal mesenchymal cell line MDA-MB-231, followed by the basal epithelial cell line MDA-MB-468, the HER2-OE cell line SK-BR-3, and the luminal A cell line MCF-7 (Fig. 1C). Since the basal cell lines are normally considered to be more malignant than the luminal A cell lines^32,36,37^, this result suggested that the CD44/CD24 ratio might be a partially quantitative indicator that could evaluate cell stemness. Similarly, ALDH1 was highly expressed in the most malignant cell line MDA-MB-231 while moderately expressed in MCF-7 and MDA-MB-468 that were less malignant (Fig. 1D,E and Supplementary Figure S1), which was consistent with the previously reported findings showing the correlation of ALDH1 with tumor malignancy and poor prognosis^37,40^. Our results, together with other reports^19,37,39,40^, suggest that high CD44/CD24 ratio and ALDH1^+^ are indicators for breast cancer malignancy. However, different expression of ALDH1 and CD44/CD24 in the same cell lines was observed. For instance, in the HER2-OE cell line SK-BR-3, the CD44/CD24 ratio was low (Fig. 1C), whereas the expression level of ALDH1 was high (Fig. 1E), suggesting that single CSC marker alone might not be enough to characterize tumor stemness or to evaluate tumor malignancy and prognosis. Different CSC markers might have different functions in tumor progression and invasion. To test this hypothesis, we investigated the role of CD44/CD24 ratio and ALDH1^+^ in the proliferation, tumorigenesis, migration and metastasis of breast cancer.

### High CD44/CD24 ratio correlates with strong proliferative capacity and tumorigenicity

**Figure 2:**
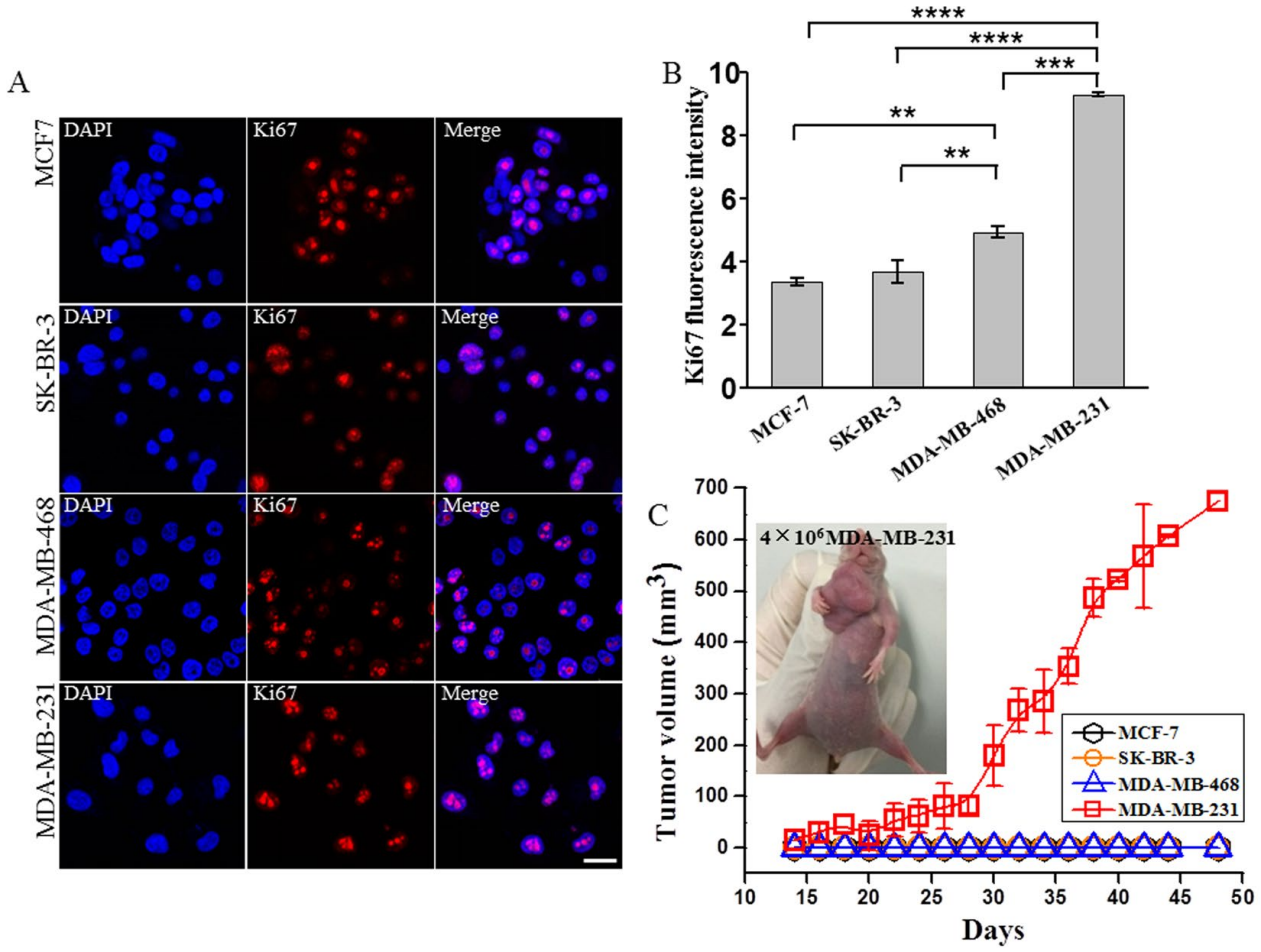
CD44/CD24 ratio positively correlates with cell proliferation and tumorigenesis. (**A**,**B**) The cell proliferation potential of different breast cancer cell lines as indicated by the expression of Ki67. Cells with high CD44/CD24 ratio exhibit high proliferation potential. (**A**) Immunofluorescence images showing the expression of Ki67 in different breast cancer cell lines. Cells were labeled with anti-Ki67 (red) and DAPI (blue), scale bar: 20 μm. (**B**) The expression level of Ki67 in in different breast cancer cell lines. The average fluorescence intensities were calculated from three parallel immunofluorescence images. Data represent means ± SD (n = 3), **P < 0.01, ***P < 0.001, ****P < 0.0001. (**C**) Breast cancer cells with high CD44/CD24 ratio exhibit strong tumorigenic ability. Tumor growth curved of the four subtypes of breast cancer cells injected into female BALB/c nude mice at the amount of 4 × 10^6^ cells. Data represent means ± SD (n = 3).

**Figure 3:**
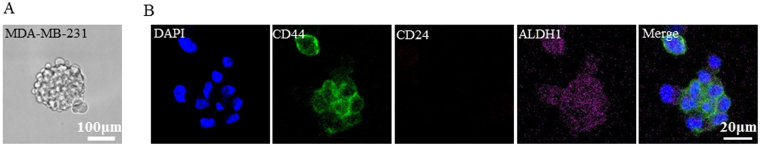
The expression of CD44, CD24, and ALDH1 in mammosphere formed by MDA-MB-231 cells. (**A**) Optical microscope image showing MDA-MB-231 cells growing as non-adherent mammospheres in medium after 10 d of cultivation. Scale bar, 100 μm. (**B**) Representative immunofluorescence images showing the expression of CD44, CD24 and ALDH1 in mammosphere formed by MDA-MB-231, in accordance with the expression of cells. Cells were labeled with anti-CD44 (green), anti-CD24 (red) and anti-ALDH1 (magenta), the nuclei stain DAPI (blue). Scale bar: 20 μm.

To investigate the relationship between CSC markers and the proliferative capacity of tumor, we first examined the proliferative capacity of the four breast cancer cell lines by checking the expression of antigen Ki67 that was an indicator normally used for proliferative capacity (Fig. 2A)^41^. Quantitative analysis of the immunostaining fluorescence images showed that the expression level of Ki67 was the highest in the basal mesenchymal cell line MDA-MB-231, followed by the basal epithelial cell line MDA-MB-468, the HER2-OE cell line SK-BR-3, and the luminal A cell line MCF-7 in turn (Fig. 2B). The trend was exactly the same with the CD44/CD24 ratio in these cell lines (Fig. 1C), indicating that CD44/CD24 ratio is positively correlated with the proliferative capacity of cells. We further investigated whether CD44/CD24 ratio was also correlated with the tumorigenesis of breast cancer cells by comparing the tumorigenicity of the four subtypes of cell lines in the xenotransplanted models. Each cell line was injected independently into the mammary fat pads of female BALB/c nude mice. When the injected cell number was 4 × 10^6^/mouse, only the mice injected with MDA-MB-231 cells with the highest CD44/CD24 ratio could generate tumors, while the other three cell lines could not (Fig. 2C and Supplementary Figure S2A). This was as expected since the cells with high CD44/CD24 ratio were demonstrated to have stronger proliferative and self-renewal capacity (Fig. 2A). The volume of the xenografted tumor increased gradually and reached ~670 mm^3^ after 48 days (Fig. 2C). When the number of the injected cells was increased to 8 × 10^6^/mouse, MCF-7 and SK-BR-3 that had the lowest CD44/CD24 ratio still could not form tumors, while tumor growth was observed for mice injected with MDA-MB-468 cells bearing the moderate CD44/CD24 ratio, though the tumor growth was much slower than MDA-MB-231 ones: the size of the generated tumor reached 93.21 mm^3^ for MDA-MB-468 after 48 days, compared to 670 mm^3^ for MDA-MB-231 at the same stage (Supplementary Figure S2B, S2C). These results indicated that high CD44/CD24 ratio was highly associated with the proliferative capacity and the tumorigenicity of breast cancer, suggesting that CD44/CD24 is a powerful CSC marker for breast cancer. This was further proved by the mammosphere formation assay that has been commonly used to test the stem-like characteristics of cancer cells^42^. After 10 days of cell culture, mammospheres were observed by optical microscope. MDA-MB-231 cells were found to form tightly packed mammospheres with the average diameter of 160 μm (Fig. 3A). By comparison, the mammospheres derived from SK-BR-3 and MDA-MB-468 cells were small (average diameter 60 μm) and loose, while MCF-7 cells were only able to form clusters containing several cells (Supplementary Figure S3), indicating stronger stem-like characteristics of MDA-MB-231 cells compared to the other three cell lines. We further stained the mammospheres derived from MDA-MB-231 cells by fluorescent antibodies against CD44, CD24, and ALDH1, and found that CD44 and ALDH1 were highly expressed in the mammospheres while no expression of CD24 was observed under fluorescence microscope (Fig. 3B and Supplementary Figure S4). These results further verified the validity of CD44/CD24 as a breast cancer CSC marker.

### ALDH1^+^ correlates with tumor metastatic capacity

**Figure 4:**
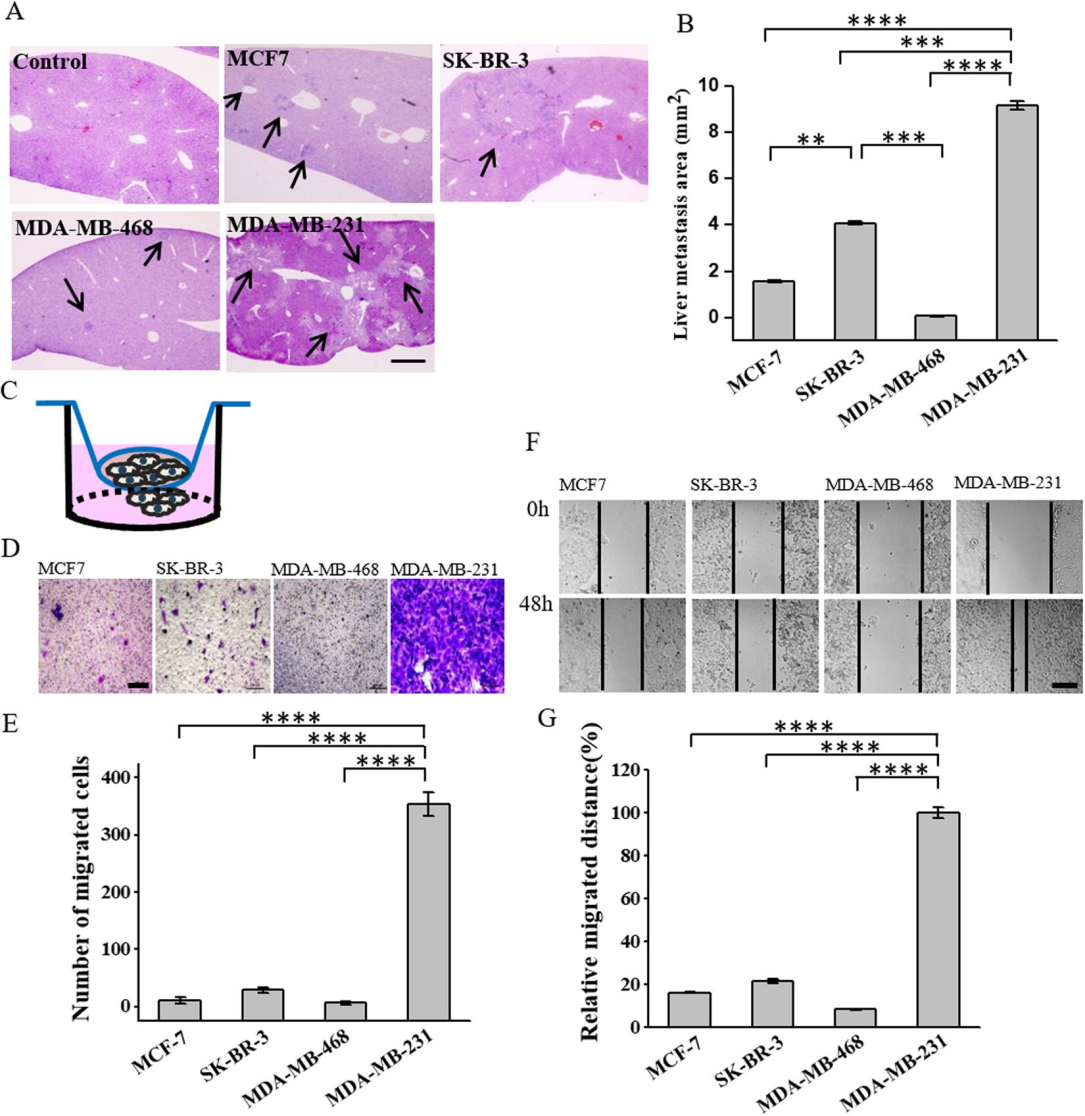
ALDH1 expression positively correlates with cell migration and tumor metastasis. (**A**) High ALDH1 expression promotes liver metastasis of breast cancer. Representative hematoxylin and eosin (H&E) staining images of livers isolated from mice six weeks after being injected with different subtypes of breast cancer cells. Mice injected without cells were used as control. Scale bar, 1mm. (**B**) The calculated liver metastasis area of mice injected with breast cancer cell lines. Data represent means ± SD (n = 3), **P < 0.01, ***P < 0.001, ****P < 0.0001. (**C**–**E**) Transwell migration assay. (**C**) Schematic illustration of the transwell migration assay. Transwell chambers with 8-μm-pore polycarbonate membranes were placed in a 24 well plate, 2 × 10^5^ of cells were seeded in the upper chamber of the assay model. Cells migrated to the bottom chamber were counted after 24h by staining with crystal violet. (**D**) The representative microscope images of the cells migrated to the bottom chamber, scale bar, 200 μm. (**E**) The average number of migrated cells in different breasts cancer cell lines (n = 3), ****P < 0.0001. (**F**,**G**) Wound healing assay. (**F**) Representative images showing the changes of wounds of the four breast cancer cells in the six-well culture plate. Images were obtained at 0 and 48 hours after the creation of the wounds. Scale bar 200 μm. (**G**) The quantification of cell migration distance in the four breast cancer cells. Data represent means ± SD (n = 3), ****P < 0.0001.

We then investigated the relationship between CSC markers and the metastatic capacity of breast cancer by checking the metastasis of the four subtypes of breast cancer cell lines in the xenotransplanted mice. Different subtypes of cells were implanted independently into the mammary fat pads of female BALB/c nude mice at the concentration of 8 × 10^6^/mouse. The mice were euthanized 48 days after injection. Their livers were extracted and stained with hematoxylin-and-eosin (H&E) that has been commonly used for the histological examination of tumor in tissue sections^43^. We observed that all the four subtypes of breast cancer cells, including MCF-7 and SK-BR-3 that were not able to generate tumors in the immunodeficient mice, formed metastasis in the liver (Fig. 4A). The size of the metastatic area in the liver was the largest (~9.1327 mm^2^) with MDA-MB-231 (Fig. 4A,B), indicating that this cell line had the strongest metastatic capacity. Given that MDA-MB-231 was also found to have the strongest proliferative and tumorigenic capacity (Fig. 2), this cell line seemed to be more aggressive compared to the others, which was in accordance with the previous findings showing that patients with the triple-negative breast cancer had higher metastatic potential and poorer prognosis^44^. Interestingly, MDA-MB-468 that had the second strongest proliferative and tumorigenic capacities did not form metastasis more easily than the cell lines with lower proliferative and tumorigenic capacities. On the contrary, the HER2-OE subtype SK-BR-3 had much larger metastatic area (~4 mm^2^), followed by the luminal A subtype MCF-7 (~1.566 mm^2^), while MDA-MB-468 had the smallest metastatic area (~0.064 mm^2^) (Fig. 4B). This trend was more similar to that with the expression level of ALDH1 compared to CD44/CD24 in the cell lines (Fig. 1E), indicating that the expression level of ALDH1, rather than CD44/CD24, is positively correlated with the metastatic capacity of breast cancer. We further performed transwell and wound healing assays to investigate the migration capability of the cells. In the transwell assay, 2 × 10^5^ of each subtype of breast cancer cells were seeded to the upper chamber, and the cells migrated to the lower chamber were counted after 24 hours (Fig. 4C). We found that MDA-MB-231 that had the highest expression level of ALDH1 exhibited significantly higher level of random migration, while MCF-7 and MDA-MB-468 that had low expression level of ALDH1 showed small fraction of migration (Fig. 4D,E). This was in accordance with the results from the wound healing assay showing that the migration distance of MDA-MB-231 was six fold that of MCF-7 and twelvefold that of MDA-MB-468 (Fig. 4F,G). These results demonstrated the positive correlation between ALDH1 and cell migration, further explained the high metastatic capacity of ALDH1^+^ cells. We noticed that though SK-BR-3 and MDA-MB-231 had similar expression level of ALDH1 (Fig. 1D,E), the tumor metastatic and the cell migration capabilities of SK-BR-3 were much lower than those of MDA-MB-231 (Fig. 4A,B and D–G). This might be because that SK-BR-3 had lower CD44/CD24 ratio compared to MDA-MB-231 (Fig. 1C), which might also contribute to slower tumor migration and metastasis. Also, the CD44/CD24 ratio was essential for tumor growth (Fig. 2). This might result in slower proliferation of tumor cells in the liver metastasis in SK-BR-3.

**Figure 5:**
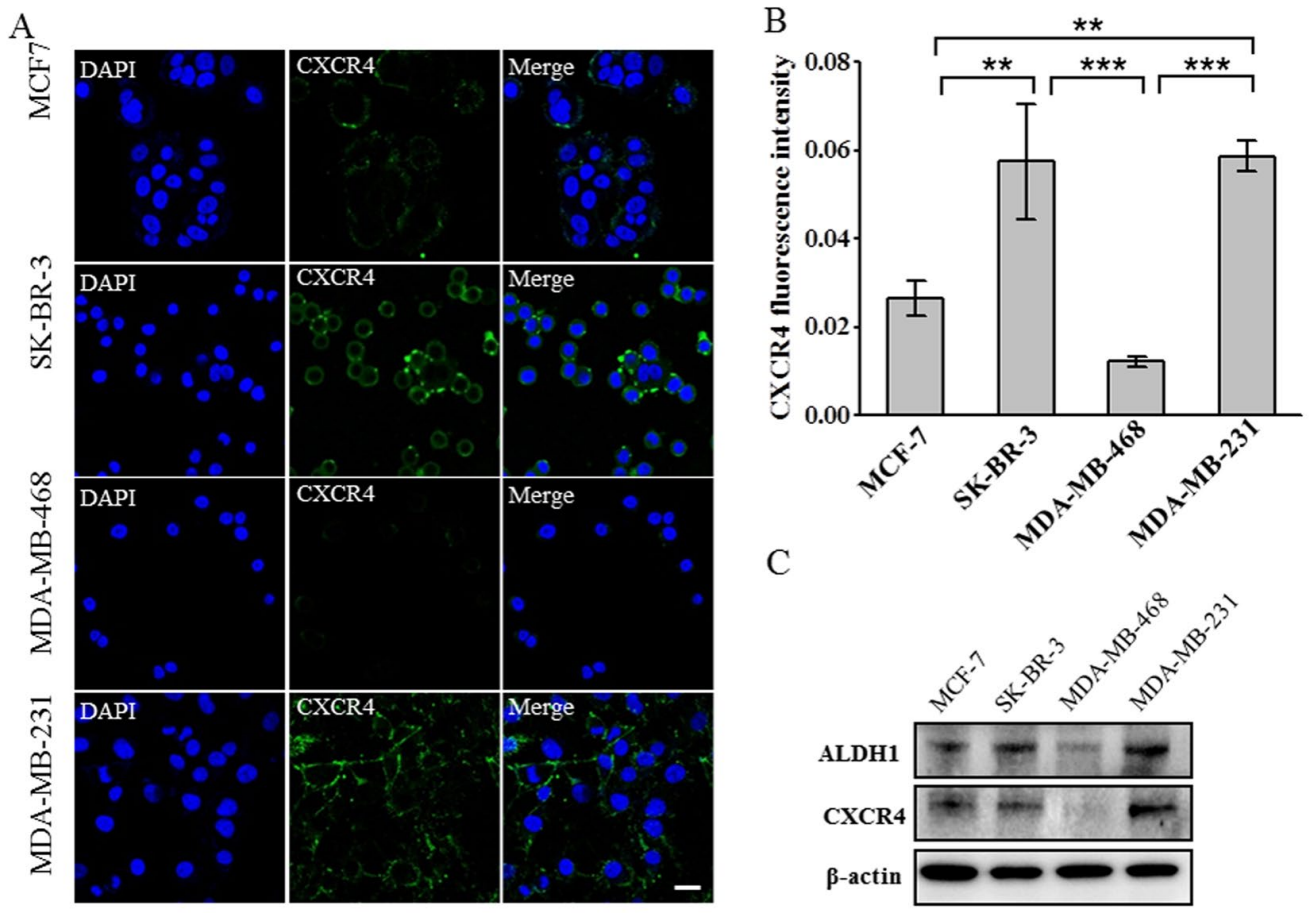
ALDH1 expression positively correlates with the expression of the tumor metastatic marker CXCR4. (**A**) Immunofluorescence images showing the expression of CXCR4 in different breast cancer cell lines. Cells were labeled with anti-CXCR4 (green) and DAPI (blue), scale bar: 20 μm. (**B**) The expression level of CXCR4 in in different breast cancer cell lines. The average fluorescence intensities were calculated from three parallel immunofluorescence images. Data represent means ± SD (n = 3), **P < 0.01, ***P < 0.001. (**C**) Western blot analysis of the expression of ALDH1, CXCR4 in different breast cancer cell lines. β-actin was used as control. The bolts were cropped from their original images and the full-length blots were presented in Supplementary Figure S5.

We also measured the expression of a G-protein coupled receptor, C-X-C chemokine receptor 4 (CXCR4) that is a key mediator in the cross-talking between tumor cells and their microenvironment, and is overexpressed in more than 20 kinds of tumors^45^. It selectively binds to the C-X-C chemokine stromal-derived factor-1 (SDF-1 or CXCL12), leading to the activation of various intracellular signaling transduction pathways and the relevant cellular behaviors, such as chemotaxis, migration, adhesion, invasion and infiltration^46^. As expected, immunostaining revealed that CXCR4 was highly expressed in MDA-MB-231 and SK-BR-3 cell lines, moderately expressed in MCF-7 cell line, while nearly negative in MDA-MB-468 cell line (Fig. 5A,B), positively correlated with the expression level of ALDH1 in these cell lines (Fig. 2). These findings were further verified by Western blot (Fig. 5C). As CXCR4 has been considered to mediate the trafficking and metastasis of cancer cells, especially the cancer stem cells^47^, these results further suggested that ALDH1 might promote the dissemination and metastasis of breast cancer through CXCR4-mediated signaling pathways.

### Suppression of CD44 and ALDH1 cause decreased tumorigenicity and cell migration capacity

**Figure 6:**
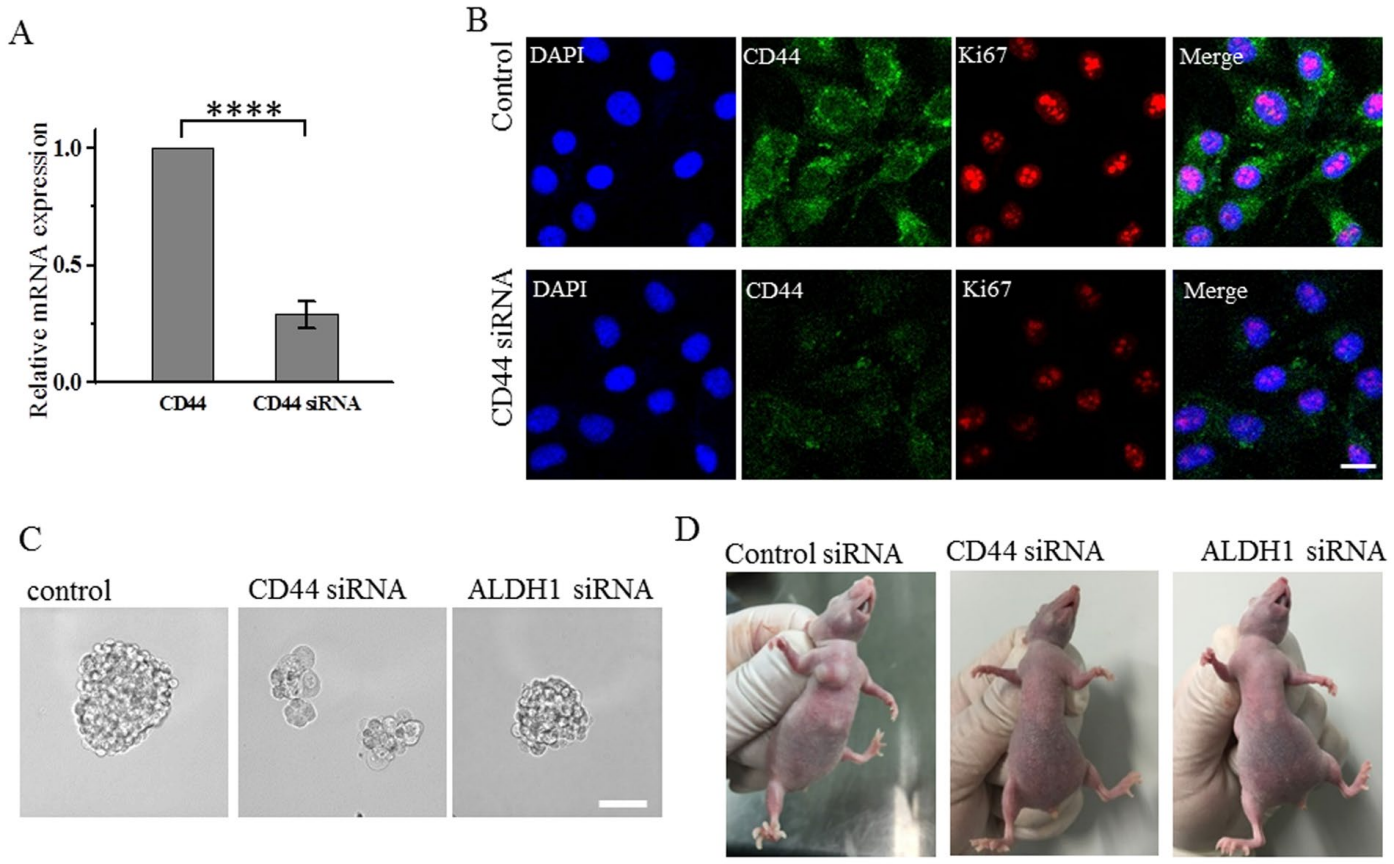
Suppression of CD44 decreases the expression of Ki67, and the capacities for mammosphere formation and tumorgenesis. (**A**) Analysis of CD44 mRNA levels following siRNA treatment. The mRNA expression decreased to 0.41 after CD44 siRNA treatment compared with control, Data represent means ± SD (n = 3), ****P < 0.0001. (**B**) Immunofluorescence images showing the expression of CD44, Ki67 in cells treated with control siRNA or CD44 siRNA, scale bars: 20 μm. (**C**) MDA-MB-231 cells transfected by control siRNA, CD44 siRNA or ALDH1 siRNA growing as non-adherent mammospheres after 10 d of mammosphere cultivation. Scale bar, 100 μm. (**D**) MDA-MB-231cells are tumorigenic while cells transfected by CD44 siRNA or ALDH1 siRNA could not form tumor with amount of 4 × 10^6^ in BALB/c nude mice after two weeks. All the experiments were performed in triplicate.

**Figure 7:**
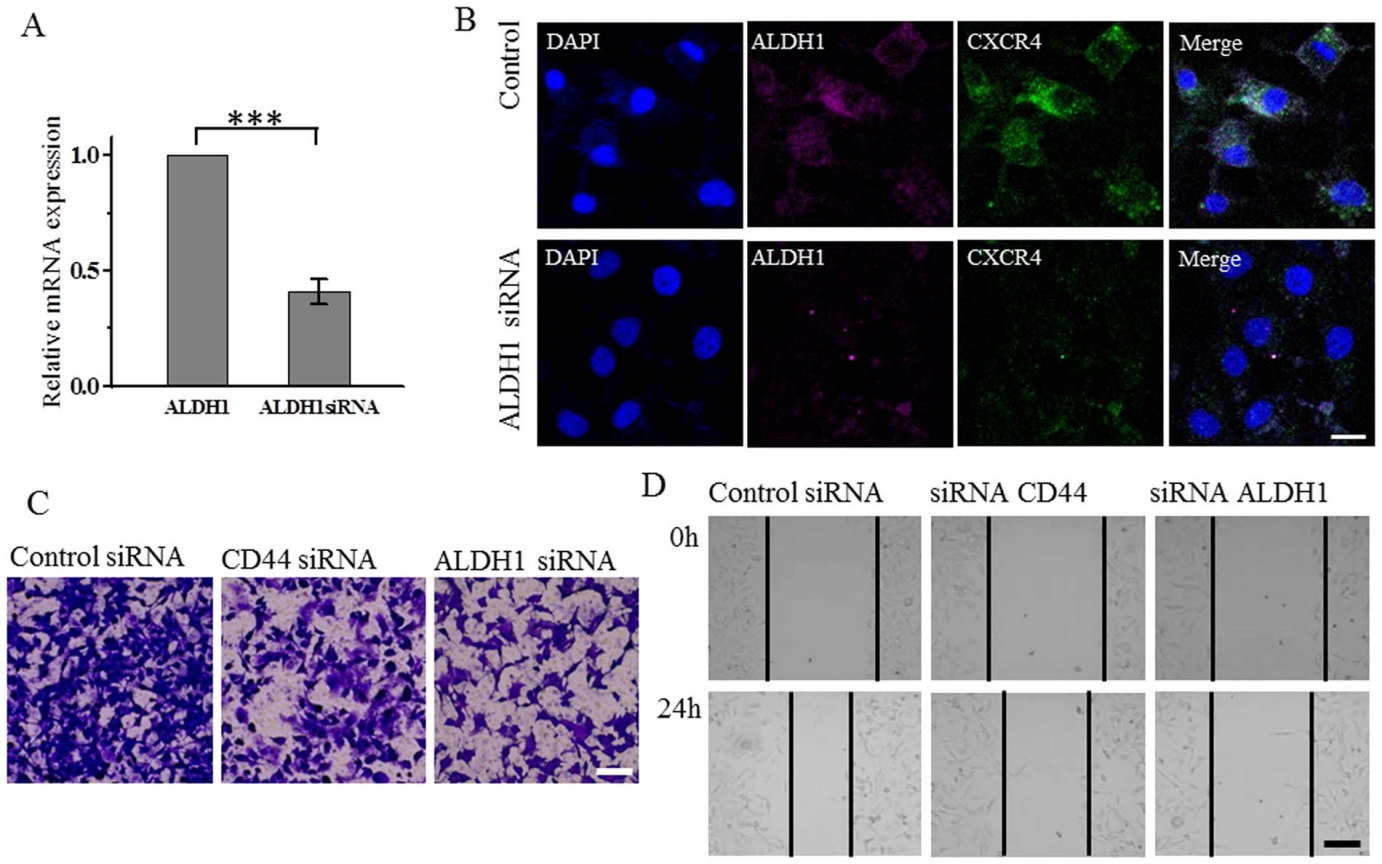
Suppression of ALDH1 decreases the expression of CXCR4 and the tumor cell migration capacity. (**A**) Analysis of ALDH1 mRNA levels following siRNA treatment. The mRNA expression decreased to 0.29 after ALDH1 siRNA treatment compared with control, Data represent means ± SD (n = 3), ***P < 0.001. (**B**) Immunofluorescence images showing the expression of ALDH1, CXCR4 in cells treated with control siRNA or CD44 siRNA, scale bars: 20 μm. (**C**) Transwell migration assay. Representative microscope images of the MDA-MB-231 cells transfected by CD44 or ALDH1 siRNA migrated to the bottom chamber, scale bar, 200 μm. (**D**) Wound healing assay. Representative images showing the changes of wounds of the MDA-MB-231 cells transfected by CD44 or ALDH1 siRNA in the six-well culture plates. Images were obtained at 0 and 24 hours after the creation of the wounds. Scale bar 200 μm. All the experiments were performed in triplicate.

After demonstrating that high CD44/CD24 ratio correlated with proliferation and tumorogenesis while ALDH1^+^ correlated with tumor metastasis, we further verified the roles of high CD44/CD24 ratio and ALDH1^+^ by suppressing their expression in MDA-MB-231 cells using siRNA. As we expected, immunostaining showed that after suppressing CD44, the expression of Ki67 significantly decreased (Fig. 6A,B), suggesting that the suppression of CD44 caused reduced proliferative capacity of cells. This was further confirmed by the mammosphere formation assay (Fig. 6C) and the tumorigenesis in the xenotransplanted mice (Fig. 6D). Mammosphere formation assay showed that after 10 days of cell culture, MDA-MB-231 cells transfected with CD44 siRNA formed no non-adherent mammosphere, while MDA-MB-231 cells transfected with ALDH1 siRNA formed tightly packed mammospheres similar to those formed by MDA-MB-231 cells treated with control siRNA, though the size of the mammospheres was slightly smaller (the average diameter of mammospheres formed by MDA-MB-231 cells transfected with ALDH1 siRNA, was 110 μm, compared to the average diameter of mammospheres formed by MDA-MB-231 cells transfected with control siRNA that was 170 μm) (Fig. 6C). These results indicated that both CD44 siRNA and ALDH1 siRNA could affect the mammosphere formation of MDA-MB-231 cells, though the influence of CD44 siRNA is stronger. We then investigated the tumorigenicity of MDA-MB-231 cells after suppressing CD44 and ALDH1 by injecting MDA-MB-231 cells that were transfected with the control siRNA, CD44 siRNA or ALDH1 siRNA into the mammary fat pads of female BALB/c nude mice at 4 × 10^6^ cells/mouse. After two weeks, MDA-MB-231 cells transfected with the control siRNA could generate tumors, while MDA-MB-231 cells transfected with CD44 siRNA or ALDH1 siRNA could not (Fig. 6D), indicating that the suppression of CD44 and ALDH1 both reduced the tumorigenicity of breast cancer cells. We also performed immunostaining to investigate whether suppressing ALDH1 would affect the expression of CXCR4. As we expected, the expression of CXCR4 significantly decreased after the suppression of ALDH1 (Fig. 7A,B), indicating that the expression of ALDH1 positively correlated with that of CXCR4. We then performed transwell and wound healing assays to investigate the migration capacity of MDA-MB-231 cells after the suppression of CD44 and ALDH1. Both assays showed that the cells transfected with CD44 siRNA or ALDH1 siRNA exhibited reduced migration level compared to the cells treated with the control siRNA (Fig. 7C,D). Cells transfected with ALDH1 siRNA exhibited more reduction in cell migration than those transfected with CD44 siRNA. These results indicated that both CD44 and ALDH1 contributed to cell migration of breast cancer cells, and ALDH1 gave more contribution.

### CD44/CD24 ratio and ALDH1 expression remain stable from the primary tumor to the metastases

**Figure 8:**
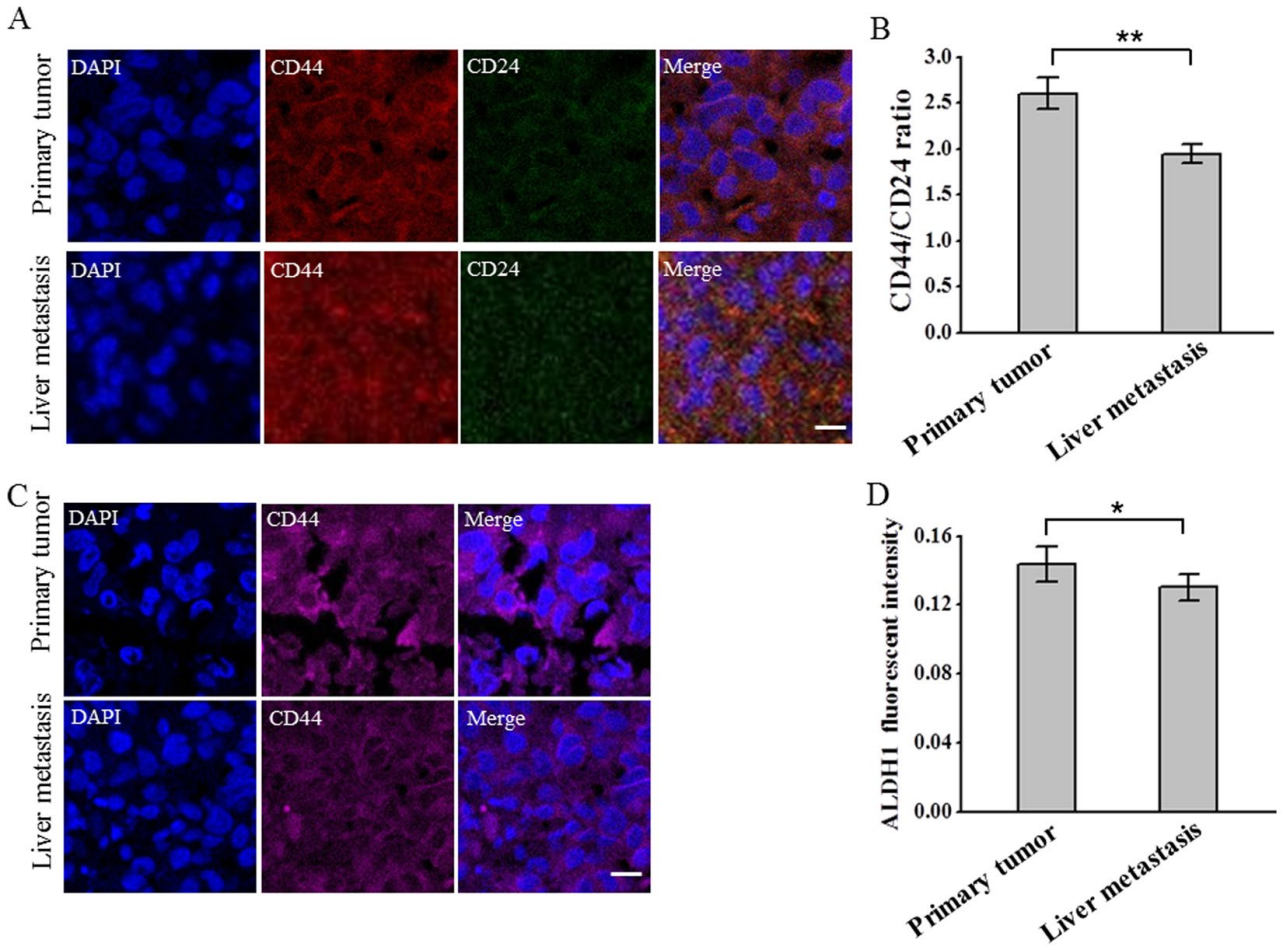
CD44/CD24 ratio and ALDH1 remain stable from the primary tumor to the liver metastases. (**A**) Representative immunofluorescent images showing the expression of CD44, CD24 in the primary tumor and liver metastases of immunodefidient mice injected with MDA-MB-231 cells. Tissues were labeled with anti-CD44 (red), anti-CD24 (green) and the nuclei stain DAPI (blue). Scale bar: 20 μm. (**B**) CD44/CD24 ratios in the primary tumor and the liver metastases. The average ratios of CD44/CD24 were calculated from three parallel immunofluorescent images. All the experiments were performed in triplicate. Data represent means ± SD (n = 3), **P < 0.01. (**C**) Representative immunofluorescent images showing the expression of ADLH1 in the primary tumor and liver metastases of immunodefidient mice injected with MDA-MB-231 cells. Tissues were labeled with anti-ALDH1 (magenta) and the nuclei stain DAPI (blue). Scale bar: 20 μm. (**D**) The average fluorescent intensities of ALDH1 from the primary tumor and the liver metastases. The average fluorescent intensities were calculated from three parallel immunofluorescent images. All the experiments were performed in triplicate. Data represent means ± SD (n = 3).

Studies have shown that CSCs are dynamic and are influenced by their surrounding microenvironment^48–50^. Although we have demonstrated the correlation between CD44/CD24 ratio, ALDH1^+^ and the development, metastasis of breast cancer, whether these CSC markers are conserved and how they are dynamically changed during tumor progression have yet to be elucidated. To this end, we tended to explore the dynamic changes of the CD44/CD24 ratio and the expression of ALDH1 during the development and metastasis of breast cancer. The primary tumor and the liver metastases of the mice burdening MDA-MB-231 cells were stained with antibodies targeting CD44, CD24 and ALDH1 and were imaged with fluorescent microscopy. We observed the expression of CD44, CD24 and ALDH1 in both the primary tumor and the metastases, indicating that these markers did not disappear during the development and metastasis of breast cancer (Fig. 8A,B). We further calculated the average fluorescent intensities of the images and found that the CD44/CD24 ratio and the expression of ALDH1 remained high, though the CD44/CD24 ratio declined slightly, indicating that these two CSC markers remained stable during metastasis (Fig. 8C,D). These results suggested the significance of CD44/CD24 and ALDH1^+^ during breast cancer progression and metastasis. The slightly positive expression of CD24 in the primary tumor and metastases might be mainly due to the complicated microenvironment of tumor, which included blood vessels and other microenvironmental cells that were CD24^+^. Besides, cellular differentiation from the cell lines to the tumors might cause the expression change of protein markers, which might also lead to CD24^+^. The slight decline in CD44/CD24 might be due to the different microenvironment between the tumor and the liver that caused the CSC phenotypic plasticity^51^.

### Circulating tumor stem cells (CTSCs) exist in tumor metastasis

**Figure 9:**
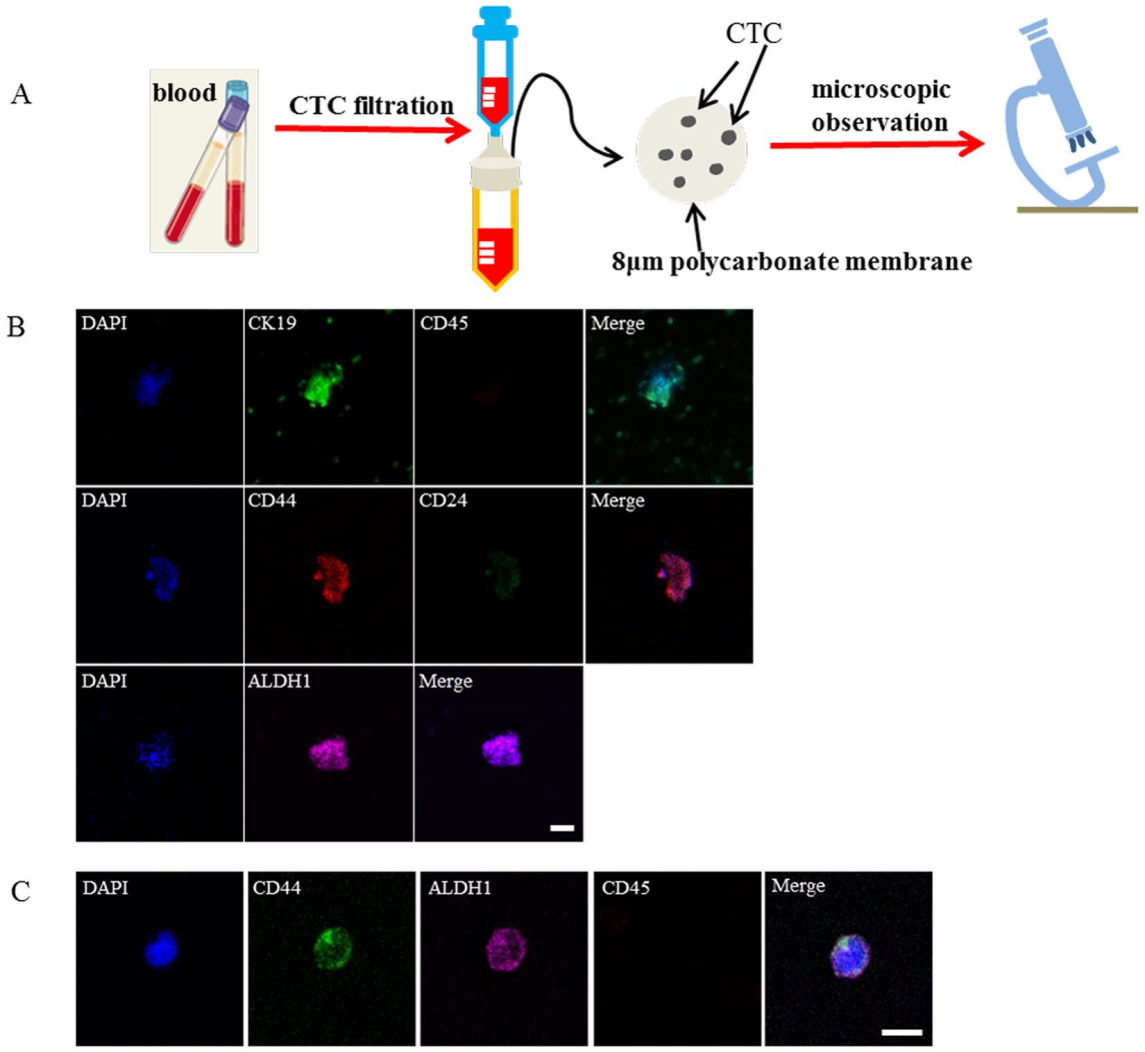
CTCs exhibit high CD44/CD24 ratio and ALDH1 expression. (**A**) Schematic illustration of the isolation and detection of CTCs from the blood using membrane filter. The isolation device contains an 8 μm pore filtering membrane and a 25 μm diameter filter holder. (**B**) Immunofluorescent images showing the expression of CK19, CD45, CD44, CD24 and ALDH1 in CTCs isolated from the immunodeficient mice injected with MDA-MB-231 cells. CTCs captured on the 8 μm polycarbonate membrane were stained with anti-CK19 (green), anti-CD45, anti-CD44 (red), anti-CD24 (green), anti-ALDH1 (magenta) and DAPI (blue), respectively. Scale bar: 20 μm. (**C**) Immunofluorescent images showing the expression of CD44, ALDH1 and CD45 in CTCs isolated from breast cancer patient. CTCs captured on the 8 μm polycarbonate membrane were stained with anti-CD44 (green), anti-ALDH1 (magenta), anti-CD45 (red), and DAPI (blue), respectively. Scale bar: 20 μm.

Since CTCs represent the primary cause of the intractable metastatic disease and are considered essential for the formation of metastasis^52^, we also explored the status of CD44/CD24 ratio and ALDH1^+^ in CTCs. CTCs were obtained from the xenotransplanted mice injected with MDA-MB-231 cells. Blood were extracted from the mice 6 weeks after cell injection. The metastasis was confirmed in the liver. CTCs were isolated from the blood by size using membrane filter (Figure 9A). The enriched cells on the filtering membranes were separated into three aliquots, and stained for CK19, CD45, CD44/CD24 and ALDH1 respectively. Immunofluorescence results revealed that the isolated cells were CK19^+^/CD45^−^ (Fig. 9B), confirming the successful isolation of CTCs from the blood. The isolated CTCs exhibited high CD44/CD24 ratio (Fig. 9B) and high expression level of ALDH1 (Fig. 9B), indicating that these two stem markers were conserved in CTCs. We further examined the existence of CSCs in CTCs from two individual cancer patients: one advanced breast cancer patient and one liver cancer patient. CTCs from the two cancer patients were collected in the same way as the immunodeficient mice. The enriched cells on the filtering membranes were stained for CD45, CD44 and ALDH1 respectively. Immunofluorescence results revealed that the isolated CTCs from the advanced breast cancer patient exhibited high expression of CD44 and ALDH1 (Fig. 9C), indicating the conservation of these two stem markers in CTCs from breast cancer patients. Interestingly, CD44 and ALDH1 were also highly expressed in CTCs from the liver patient (Supplementary Figure S6), suggesting that these two markers may be also conserved in CTCs in liver cancer. These results, together with the conservation of high CD44/CD24 ratio and ALDH1^+^ in both the primary tumor and the metastases, suggested the importance of CSCs during tumor progression and metastasis. Moreover, unlike many epithelial markers, such as EpCAM and CK19 that usually disappear during circulation and metastasis as a result of epithelial to mesenchymal transition (EMT)^53–55^, these conserved CSC markers hold a great potential as the reliable biomarkers for monitoring cancer progression and predicting prognosis.

## Discussion

In this study, we investigated the expression of CD44, CD24 and ALDH1 in different subtypes of breast cancer, and explored the correlation between them and cancer progression both *in vitro* and *in vivo*. We found that high CD44/CD24 ratio and ALDH1^+^ were related to cancer malignancy. However, they performed different functions in tumor progression. High CD44/CD24 ratio was mainly in charge of self-renewal, proliferation, and tumor growth, while ALDH1^+^ represented a stronger capability for invasion and metastasis. The CD44/CD24 ratio and ALDH1 level were stable in the primary tumor and the distant metastases, and existed in CTCs, indicating the conservation of these two stem markers during the progression and metastasis of breast cancer. These results demonstrated the potential of these CSC markers in monitoring tumor progression and predicting prognosis.

The combination of the transmembrane proteins CD44 and CD24 has been used to characterize the stemness of cancer cells over a long period of time. Ever since Al-Hajj *et al*. reported that CD44^+^/CD24^−/low^ cells were more tumorigenic than CD44^+^/CD24^+^ cells in the breast cancer^7^, CD44^+^/CD24^−/low^ has been widely accepted as a CSC marker and predictor for the prognosis of breast cancer^15,16^. Here we found that the CD44^+^/CD24^−^ cell line MDA-MB-231 definitely had remarkably stronger proliferative and tumorigenic capacities compared to the CD44^+^/CD24^+^ cell line MDA-MB-468 and the CD44^−^/CD24^+^ cell lines MCF-7 and SK-BR-3. However, CD44/CD24 ratio seemed to be a more effective way to evaluate the stem characteristics of cancer cells compared to CD44^+^/CD24^−^, as for the non CD44^+^/CD24^−^ cell lines (MDA-MB-468, CD44^+^/CD24^+^; MCF-7 and SK-BR-3, CD44^−^/CD24^+^, Fig. 1A–C), MDA-MB-468 that has higher CD44/CD24 ratio exhibited much stronger proliferative and tumorigenic capacities than MCF-7 and SK-BR-3 that have lower CD44/CD24 ratio (Fig. 2).

Moreover, although various CSC markers have been widely used to characterize the stem properties of cancers and to predict the prognosis, few studies investigated the relationship between different CSC markers, and the definition of CSCs based on the expression of stem markers is vague. Here we found that the CD44/CD24 ratio and the expression of ALDH1 were not consistent in the breast cancer (Fig. 1), suggesting their different origins and properties. We further demonstrated that these two markers performed different functions during tumor progression and metastasis (Figs 2, 4 and 5). These results suggested that single CSC marker alone was not enough to characterize the stem properties of cancer. On the contrary, a combination of a set of CSC markers would be a more reliable way to evaluate the stem properties of tumors.

Finally, we found that the CD44/CD24 ratio and ALDH1^+^ were stable during the tumor growth and metastasis, from the primary tumor to the distant metastases (Fig. 8). As these two markers were also found to exist in CTCs (Fig. 9), one may suspect that CSCs might enter circulation and participate in tumor metastasis, though further studies need to be performed. The conservation of CD44/CD24 ratio and ALDH1^+^ during tumor progression and metastasis, especially the expression of these markers in CTCs, provided possibilities to evaluate tumor stemness through the liquid biopsy, and therefore opened a new avenue towards the early diagnosis, the tumor progression monitoring, and the prognosis evaluation of cancers.

## Materials and Methods

### Cell culture

All the cell lines were obtained from the American Type Culture Collection (ATCC) (Manassas, VA, USA). The MCF-7, SK-BR-3 and MDA-MB-231 cells were cultured in high glucose GlutaMAX(™) Dulbecco’s Modified Eagle Medium (DMEM) (GIBCO-BRL Co, MD, USA), supplemented with 10% fetal bovine serum (FBS) (GIBCO-BRL) and 1% penicillin/streptomycin (GIBCO-BRL). Cells were maintained in a humidified atmosphere of 5% CO_2_–95% air at 37 °C. MDA-MB-468 cells were cultured in L-15 medium (GIBCO-BRL) supplemented with 10% FBS and 1% penicillin/streptomycin, maintained in a humidified atmosphere of 0% CO_2_–100% air at 37 °C. Subcultivation of all the cell lines was performed using 0.25% trypsin and 5 mM ethylenediaminetetraacetic acid (EDTA) (GIBCO-BRL). Fresh primary breast tumor cells from the mice burdening MDA-MB-231 cells were collected after dissociation and digestion with trypsin at 37 °C for 20 min, and were then cultured in a humidified atmosphere of 5% CO_2_–95% air at 37 °C.

### Subject selection and blood collection

The study protocol was approved by the Medical Ethical Committee of Peking Cancer Hospital (Approval No.: 2013KT29). Patients with advanced cancer were recruited in Peking University Cancer Hospital according to an institutional board approved protocol. Signed informed consent was obtained from all patients. All experiments were performed in accordance with relevant guidelines and regulations. The blood drawn by venous puncture was collected in the anticoagulative blood collection tubes. Red cells were removed using Red Blood Cell Lysis Buffer before CTCs isolation.

### Flow cytometry analysis

For flow cytometry analysis, the breast cancer cells at the logarithmic growth phase were digested with 0.25% trypsin and washed with PBS for three times, followed by being re-suspended in 100ul PBS, and then stained with anti-CD44-PE and anti-CD24-FITC or stained with their isotype controls at room temperature for 40 min. The samples were then washed by PBS three times and finally re-suspended in 200 μl PBS. Flow cytometry analysis was performed on a BD Accuri^TM^ C6 Flow Cytometer (BD Bioscience). The expression ratio of CD44 and CD24 (CD44/CD24) in different subtypes of breast cancer cell lines were calculated from the percentage of CD44 and CD24 positive subpopulations in the flow cytometry analysis.

### ALDEFLUOR assay

The ALDEFLUOR kit (StemCell Technologies, Durham, NC, USA) was used to analyze the population with high ALDH enzymatic activity according to the manufacturer’s instructions. Briefly, the cells were incubated in the ALDEFLUOR assay buffer containing ALDH substrate (BAAA, 1 mmol/l per 1 × 10^6^ cells), and incubated for 40 min at 37 °C. In each experiment, an aliquot of cells was incubated under identical conditions, using 50 mmol/l of diethylaminobenzaldehyde (DEAB), a specific ALDH inhibitor, as a negative control.

### Immunofluorescent staining

When staining the cells *in vitro*, cell resuspension solutions were plated at the concentration of 5 × 10^4^ cells/ml on the confocol wells, fixed with 4 % PFA for 20 min and permeabilized with PBS containing 0.2 % Triton X-100 on the following day. The cells were then blocked for 30 min at 37 ^o^C with 5 % BSA, and incubated at 37^o^C for 1 hour with antibodies as follows: anti-CD44-PE antibody (Abcam), anti-CD24-FITC antibody (Abcam), anti-ALDH1-Alexa Fluor647 antibody (Abcam), anti-Ki67-Alexa Fluor647 antibody (Abcam), and anti-CXCR4 antibody (Abcam). Following that, the cells were washed three times with PBS. The cells incubated with anti-CXCR4 antibody need to be further stained with the donkey anti-goat IgG H&L second antibodies (Abcam) for 45 min at 37 ^o^C.

CTCs were isolated from the blood samples by size using membrane filter. Immnofluorescent determination of CTCs was performed by staining the cells on the filtering membrane directly with anti-cytokeratins19 and anti-CD45. Besides, the cells were also stained by CD44, CD24, and ALDH1 antibodies to investigate the expression of these stem markers.

For the immunofluorescent staining of paraffin-embedded sections, the samples were deparaffinized in xylene and rehydrated in graded alcohol. Antigen enhancement was performed by incubating the sections in the citrate buffer (pH 6) as recommended. After being blocked with 5% normal goat serum (Solarbio) for 30 min at room temperature, the samples were double stained with CD44-PE, CD24-FITC antibodies or stained with anti-ALDH1 antibody (Alexa Fluor647) solution diluted according to the manufacturer’s instruction for 1h at room temperature. Nuclei were counterstained with 4′,6-diamidino-2-phenylindole (DAPI, Invitrogen). The samples were then washed twice with PBS and mounted with anti-bleaching coverslips. All the samples were examined and photographed using a Single photon laser confocal imaging system, Zeiss 760 (Carl Zeiss).

### Mouse model

All the animal experiments were performed according to the NIH guidelines for the care and use of laboratory animals of Peking University Animal Study Committee’s requirements and were according to the protocol approved by the Institutional Animal Care. Mice were maintained under specific pathogen-free conditions and all the efforts were made to minimize animal suffering.

Female BALB/c nude mice at 6 weeks of age (initially weighing almost 16 g) were purchased from Vital River Laboratory Animal Technology Co. Ltd. Four subtypes of breast cancer cells were trypsinized using Trypsin-EDTA (0.25%) containing phenol red, washed once with PBS, re-suspended in culture medium at 4 × 10^6^ cells per 200 µl, and injected in triplicate into mammary fat pads of female BALB/c nude mice. Mice were monitored daily for 6 weeks. Tumor size was measured every two days with calipers. The tumor volume was determined by the following formula: ${\rm{V}}={\rm{L}}\times \frac{{W}^{2}}{2}$ (Car lsson *et al*., 1983). Mice were euthanized. The primary tumor, along with the heart, liver, spleen, lungs, and kidneys were collected from an individual mouse after euthanization at the end of the study (after 6 weeks), and fixed in 4% paraformaldehyde (PFA) (Sigma) at 4 °C and embedded in paraffin for further evaluation.

### Mammosphere culture

Single cell was plated in the ultralow attachment plates (Corning) at a density of 2 × 10^4^ viable cells/mL in the primary culture and 1000 cells/mL in the passages. Cells were grown in the mammary epithelial growth medium (MEGM, Lonza), supplemented with B27 (Invitrogen), 20 ng/mL EGF and 20 ng/mL bFGF (Sigma). Bovine pituitary extract was excluded. Mammospheres were collected by gentle centrifugation (1000 rpm) after 10 d.

### Transwell migration assay

The Transwell assay was applied using the transwell chamber with 8.0-μm-pore polycarbonate membrane insert (Corning). MCF-7, SK-BR-3, MDA-MB-468, and MDA-MB-231 cells were seeded into the upper chambers of the inserts (2 × 10^5^/chamber) in 200 μl serum-free medium opti-MEM at 37 °C. 200 μL of the complete DMEM medium was added in the lower chambers. After 24 h of incubation, cells migrating to lower chambers were dyed with crystal violet and counted under microscope.

### Wound healing assay

The four subtypes of cells were plated onto the 6-well plate to create confluent monolayers. A ‘‘scratch’’ with a p200 pipet tip was created by scraping the cell monolayer in a straight line. The debris was removed and the edge of the scratch was smoothed by washing the cells once with PBS and then replaced with 2 ml of medium. The dishes were placed under a phase-contrast microscope and the first image was acquired. The dishes were cultured in an incubator at 37 °C before being taken out and examined periodically. To obtain the same field during the image acquisition, markings were created to be used as reference points. For each image, the distance between either side of the scratch can be measured at certain intervals (mm). By comparing the distances from time 0 to the last time point (48 h), the migration distance of each cell was obtained.

### Protein separation and western blot analysis

Cells were cultured in 6-well plate (Corning), washed once with PBS (pH 7.4), and scraped using scraper (Fisherbrand). The suspension was lysed with 200 μl of lysing buffer supplemented with protease inhibitor cocktail and phenylmethylsulfonyl fluoride (Thermo scientific) on ice for 60 min. Protein fractions were collected by centrifugation at 15,000 rpm at 4 °C for 10 min. Sample loading was normalized according to BCA (Bicinchoninic acid) relative protein quantification (Solarbio). Proteins separated following a NuPAGE 10% Bis-Tris Gel (Thermo), wet electrophoretic transfer was used to transfer the proteins to polyvinylidene difluoride (PVDF) membranes (0.45 µm; Millipore, Bedford, MA). The membranes were blocked with 5% non-fat milk powder (BD Bioscience) in Tris-buffered saline with 0.1% Tween (TBST) for 1 hour at room temperature and then incubated with ALDH1A1 antibody (rabbit mAb, Cell Signal Technology) or CXCR4 antibody (goat mAb, Abcam) overnight at 4 °C, followed by horse-radish-peroxidase conjugated goat anti-rabbit IgG or donkey anti-goat IgG (Cell Signal Technology) respectively for 1 hour at RT in 0.5% non-fat milk powder with TBST. Visualization was performed using Image Quant LAS 4000 with an Enhanced Chemiluminescence Kit (Thermo Pierce, Waltham, MA, USA).

### RNA silencing

siRNA for CD44 and ALDH1 was designed from the sequence of the CD44 and ALDH1 gene obtained from the database of the National Center for Biotechnology Information (NCBI; Bethesda, MD, http://www.ncbi.nih.gov). The double-stranded CD44, ALDH1 siRNA and a scrambled control siRNA were purchased from Guangzhou RiboBio Co., Ltd. MDA-MB-231 cells at 50% confluence were transfected with CD44 siRNA or ALDH1 siRNA in triplicate in 2 ml of complete medium in six-well plates. Transfections were performed with 50 nM of siRNA using transfection reagent (RiboBio) according to the manufacturer’s instructions. The cells were then incubated at 37 °C in 5% CO_2_ for 48–72 hours. The cells were then harvested and processed for quantitative real-time RT-PCR, immunofluorescence, migration assay.

### RNA extraction

Total RNA was extracted from cells using Trizol Plus RNA purification kit (Life Technologies) according to the manufacture’s protocol. RNA dissolved in 10 μl of DEPC-treated water. Extracted RNA was quantified using a Nanodrop spectrophotometer (Thermo Fisher Scientific).

### Real-Time PCR

Extracted RNA was reverse-transcribed to generate first-strand cDNA (QuantScript RT Kit, TIANGEN) before qPCR. Quantitative Realtime PCR was performed on DNase-treated RNA using SuperReal PreMix Plus (SYBR Green) (TIANGEN) according to the manufacturer’s directions on a Realtime PCR System (eppendorf).

### CTCs isolation from the blood

CTCs were isolated from the blood by size using membrane filter (Millipore) with calibrated pores (diameter 8 μm) and a filter holder (25 μm) (Millipore Swinnex). Blood samples from the mouse or the advanced cancer patients (1 ml) were processed within 4 h. Firstly, the erythrocyte was removed using an erythrocyte-lysis buffer (Solarbio), then the supernatant was filtered by the membrane. After filtration, the membranes were washed with PBS, disassembled from the filtration module, and allowed to air-dry until staining.

### Statistical analysis

All the data were expressed as mean ± SD. The P value equal to or less than 0.05 was considered as statistically significant.

## Electronic supplementary material

Supplementary Information
