# Author Correction: Unraveling the roles of CD44/CD24 and ALDH1 as cancer stem cell markers in tumorigenesis and metastasis

**DOI:** 10.1038/s41598-018-22220-0

**Published:** 2018-03-06

**Authors:** Wenzhe Li, Huailei Ma, Jin Zhang, Ling Zhu, Chen Wang, Yanlian Yang

**Affiliations:** 1 CAS Key Laboratory of Standardization and Measurement for Nanotechnology, CAS Key Laboratory of Biological Effects of Nanomaterials and Nanosafety, CAS Center for Excellence in Nanoscience, National Center for Nanoscience and Technology, Beijing, 100190 P.R. China; 2 Academy for Advanced Interdisciplinary Studies, Peking University, Beijing, 100871 China; 3 University of Chinese Academy of Sciences, 19 A Yuquan Rd, Shijingshan District, Beijing, P.R. China 100049

Correction to: *Scientific Reports* 10.1038/s41598-017-14364-2, published online 23 October 2017

This original version of this Article contained errors.

In the legend of Figure 9,

‘(**A**) Schematic illustration of ISET to detect CTCs in the blood. ISET device contains an 8 μm pore filtering membrane and a 25 μm diameter filter holder.’

now reads:

‘(**A**) Schematic illustration of the isolation and detection of CTCs from the blood using membrane filter. The isolation device contains an 8 μm pore filtering membrane and a 25 μm diameter filter holder.’

In the Results section under subheading ‘Circulating tumor stem cells (CTSCs) exist in tumor metastasis’,

‘CTCs were isolated from the blood using ISET (isolation by size of epithelial tumor cells) method^53^ (Figure 9A).’

now reads:

‘CTCs were isolated from the blood by size using membrane filter (Figure 9A).’

In the Materials and Methods section,

‘CTCs were isolated from the blood samples by the ISET method^53^.’

now reads:

‘CTCs were isolated from the blood samples by size using membrane filter.’

and

‘**CTCs isolation by ISET technology**. ISET device contains a filtering membrane (Millipore) with calibrated pores (diameter 8 μm) and a filter holder (25 μm) (Millipore Swinnex). Blood samples from the mouse or the advanced cancer patients (1 ml) were processed within 4 h. Firstly, the erythrocyte was removed using an erythrocyte-lysis buffer (Solarbio), then the supernatant was filtered on the ISET device. After filtration, the membranes were washed with PBS, disassembled from the filtration module, and allowed to air-dry until staining.’

now reads:

‘**CTCs isolation from the blood**. CTCs were isolated from the blood by size using membrane filter (Millipore) with calibrated pores (diameter 8 μm) and a filter holder (25 μm) (Millipore Swinnex). Blood samples from the mouse or the advanced cancer patients (1 ml) were processed within 4 h. Firstly, the erythrocyte was removed using an erythrocyte-lysis buffer (Solarbio), then the supernatant was filtered by the membrane. After filtration, the membranes were washed with PBS, disassembled from the filtration module, and allowed to air-dry until staining.’

Finally, the original Reference 53 has been removed.

These errors have now been corrected in the HTML and PDF versions of the Article.

