# Supplementary material for: Unraveling the roles of CD44/CD24 and ALDH1 as cancer stem cell markers in tumorigenesis and metastasis
Source: Sci Rep. 2017 Oct 23;7:13856. doi: 10.1038/s41598-017-14364-2 (PMC5653849; doi:10.1038/s41598-017-14364-2)
Supplement: Supplementary file 1 — Supplementary Information [file 41598_2017_14364_MOESM1_ESM.doc]

**Unraveling the roles of CD44/CD24 and ALDH1 as cancer stem cell markers in tumorigenesis and metastasis**

Wenzhe Li^1,2,3^, Huailei Ma^1,3^, Jin Zhang^2^, Ling Zhu^1,3^*, Chen Wang^1,3^*, Yanlian Yang^1,3^*

^1^CAS Key Laboratory of Standardization and Measurement for Nanotechnology, CAS Key Laboratory of Biological Effects of Nanomaterials and Nanosafety, CAS Center for Excellence in Nanoscience, National Center for Nanoscience and Technology, Beijing 100190, P. R. China

^2^Academy for Advanced Interdisciplinary Studies, Peking University, Beijing 100871, China

^3^University of Chinese Academy of Sciences, 19 A Yuquan Rd, Shijingshan District, Beijing, P. R. China 100049

*Correspondence to [zhul@nanoctr.cn (Ling](mailto:zhul@nanoctr.cn (Ling) Zhu), [wangch@nanoctr.cn (Chen](mailto:wangch@nanoctr.cn (Chen) Wang), [yangyl@nanoctr.cn](mailto:yangyl@nanoctr.cn) (Yanlian Yang)

**Supplementary Table S1.** The percentages of CD44 and CD24 positive subpopulations in different subtypes of breast cancer cell lines from the flow cytometry analysis.

|  | CD44(%) | CD24(%) |
| --- | --- | --- |
| MCF7 | 2.6 | 81.7 |
| SK-BR-3 | 0.4 | 98.6 |
| MDA-MB-468 | 99.9 | 100 |
| MDA-MB-231 | 99.9 | 0.8 |


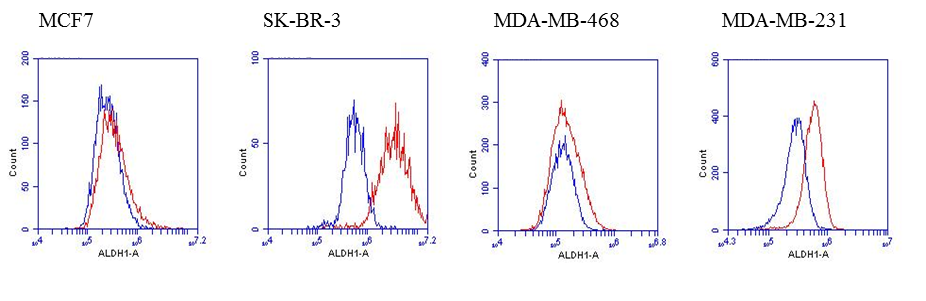


**Supplementary Figure S1. Flow cytometry analysis of the expression of ALDH1 in different molecular subtypes of breast cancer cell lines measured by AlDEFLUOR kit.** The percentage of ALDH1^+^ subpopulation was measured by incubating cells with ALDEFLUOR substrate BAAA(red), and the specific inhibitor of ALDH, DEAB, were used to establish the baseline fluorescence of these cells (blue).


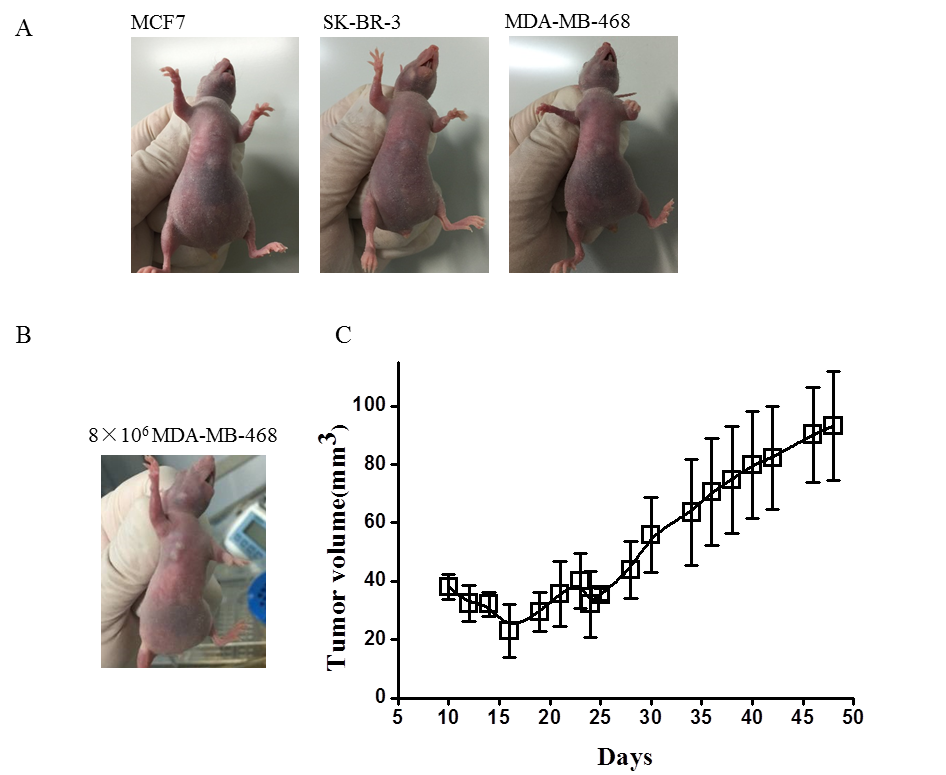


**Supplementary Figures S2. The tumorigenic ability of different subtypes of breast cancer cell lines.** (A) Representative photos showing the tumor growth of MCF-7, SK-BR-3, and MDA-MB-468 48 days after being implanted into the BALB/C nude mice at the injection amount of 4×10^6^ cells. (B) Representative photo showing the tumor growth of MDA-MB-468 48 days after being implanted into the BALB/C nude mice at the injection amount of 8×10^6^ cells. (C) Tumor growth curved of the f MDA-MB-468 injected into female BALB/C nude mice at the amount of 4×10^6^ cells. Data represent means ± SD (n=3).


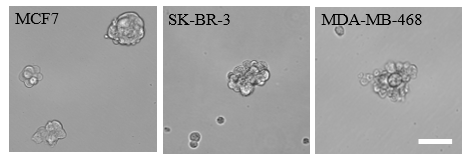


**Supplementary Figure S3. Morphology of mammospheres formed by MCF-7, SK-BR-3, and MDA-MB-468 cells, respectively.** Cells were cultured for 10 days before being collected. The SK-BR-3 and MDA-MB-468 mammospheres are small and loose, while the MCF-7 can only form clusters containing several cells. Scale bar, 100 μm.


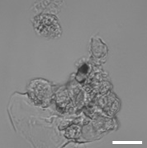


**Supplementary Figure S4.** Bright field image of mammosphere formed by MDA- MB - 231 cells under the fluorescence microscope. Scale bar, 20 μm.


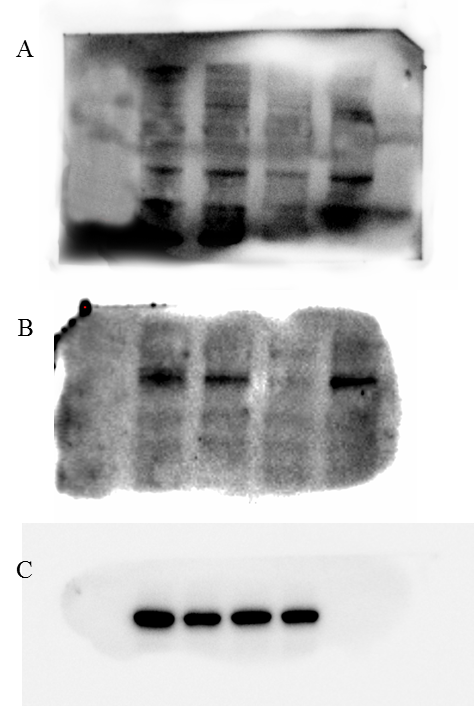


**Supplementary Figure S5** Uncropped full-length blots of (A) ALDH1, (B) CXCR4 and (C) β-actin, using Western blot measurement. (The bands are MCF-7, SK-BR-3, MDA-MB-468, and MDA-MB-231, respectively, from left to the right,.)


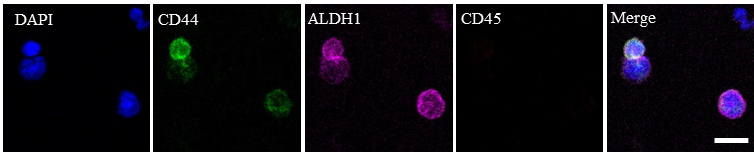


**Supplementary Figure S6** Immunofluorescent images showing the expression of CD44, ALDH1 and CD45 in CTCs isolated from liver cancer patient. CTCs captured on the 8 μm polycarbonate membrane were stained with anti-CD44 (green), anti-ALDH1 (magenta), anti-CD45 (red), and DAPI (blue), respectively. Scale bar, 20 μm.
